# Sex Hormones, Gonadotropins, and Sex Hormone-binding Globulin in Infants Fed Breast Milk, Cow Milk Formula, or Soy Formula

**DOI:** 10.1038/s41598-017-04610-y

**Published:** 2017-06-28

**Authors:** Xin Fang, Lei Wang, Chunhua Wu, Huijing Shi, Zhijun Zhou, Scott Montgomery, Yang Cao

**Affiliations:** 1 Unit of Biostatistics, Institute of Environmental Medicine, Karolinska Institutet, Stockholm, 17177 Sweden; 2 Department of Oral & Maxillofacial-Head & Neck Oncology, the Ninth People’s Hospital, Shanghai Jiao Tong University School of Medicine, Shanghai Key Laboratory of Stomatology, Shanghai, 200011 China; 3 School of Public Health/Key Laboratory of Public Health Safety of Ministry of Education, Fudan University, Shanghai, 200032 China; 4 Collaborative Innovation Center of Social Risks Governance in Health, Fudan University, Shanghai, 200032 China; 5 Clinical Epidemiology and Biostatistics, School of Medical Sciences, Örebro University, Örebro, 70182 Sweden; 6 Clinical Epidemiology Unit, Karolinska University Hospital, Karolinska Institutet, 17177 Stockholm, Sweden; 7 Department of Epidemiology and Public Health, University College London, London, WC1E 6BT UK

## Abstract

Measurement of endogenous hormones in early life is important to investigate the effects of hormonally active environmental compounds. To assess the possible hormonal effects of different feeding regimens in different sample matrices of infants, 166 infants were enrolled from two U.S hospitals between 2006 and 2009. The children were classified into exclusive soy formula, cow milk formula or breast milk regimens. Urine, saliva and blood samples were collected over the first 12 months of life. Estradiol, estrone, testosterone, luteinizing hormone (LH), follicle-stimulating hormone (FSH) and sex hormone-binding globulin (SHBG) levels were measured in the three matrices. Lower estradiol and LH levels were found in urine and saliva samples of soy formula-fed boys compared to cow formula-fed boys. Higher LH level was found in urine samples of soy formula-fed girls compared to cow formula-fed girls. However, we found neither a neonatal testosterone rise in the boys nor a gender-specific difference in testosterone levels, which suggests that urinary testosterone levels may not accurately reflect blood levels during mini-puberty. Nevertheless, our study shows that blood, urine and saliva samples are readily collectible and suitable for multi-hormone analyses in children and allow examination of hypotheses concerning endocrine effects from dietary compounds.

## Introduction

Hormonally active environmental compounds, so called “endocrine disruptors”, may be among the causes of pubertal disorders^1^, obesity^2, 3^, cryptorchidism^4^, and a variety of other conditions in childhood^5^. A key aspect of such investigations might be the measurement of endogenous hormones in early life. However, collecting multiple biological samples in well children is usually difficult; most studies reporting hormone levels have few samples, involved hospitalized children, or use pooling or other methods^6^.

An estimated 25% of infant formula sold in the United States is based on soy protein^7^. Soy formula is only clearly indicated for children with galactosemia or lactose intolerance, but it has been used in children with a variety of feeding problems for more than 60 years^8^. Soy infant formula contains plant isoflavones, mostly genistein and daidzein that have been shown to act as estrogens in experimental studies. They might prolong the effect of maternal estrogen, or interfere with hormonal homeostasis in children^9, 10^. An infant exclusively fed soy formula receives the estrogenic equivalent of between 0.01 and >1 birth control pills per day, depending on the potency estimate used for converting genistein and daidzein to estrogen equivalents^11–13^. By contrast, almost no phytoestrogens have been detected in dairy-based infant formula or in human milk, even when the mother consumes soy products, and endogenous estrogen, while appearing in breast milk, does so at low concentration^14, 15^. Although taken from dairy cows during pregnancy, the quantity of estrogens in various kinds of milk is too low (usually less than 10 pg/ml) to demonstrate biological activity^16^.

However, longitudinally collecting data on hormone levels in human infants based on feeding methods is lacking. There is only one long term follow-up study of infants fed soy that followed 811 subjects (85% of the initial study cohort) in their twenties or early thirties who, as infants enrolled in the prospective study, had been given soy formula (120 males and 128 females) or cow milk formula (295 males and 268 females)^17^. The study conducted interviews over telephone. Those given soy formula did not differ from those given cow milk based formula on their answers to general questions about health and reproduction. However, women who had been fed soy formula as infants reported longer duration of menstrual bleeding (about 8 hours) and greater discomfort with menstruation; they also reported more use of asthma or allergy drugs and a greater tendency for sedentary activities. The study found little or no evidence of excess morbidity among the women given soy as infants, nor did it find large differences in measures plausibly related to reproductive function, such as menstrual cycle length^17^. The result was criticized, however, because it did not measure hormone levels or reproductive function of individuals directly^18^.

Although it may not reflect concentrations at the site of action, urine concentrations were usually conveniently collected from infants for hormone data. Therefore, most hormone data from infants are from urine concentrations for the convenience of collection. Here, the intention was to estimate hormone levels of infants fed by different methods and also to investigate the degree to which urinary concentration reflects blood or saliva concentration. The later matrices may represent concentration at the site of action more closely than urine does^19^.

In a partly cross-sectional, partly longitudinal study to investigate possible hormonal effects for different infant feeding regimens, we collected urine, saliva, and blood from infants of different ages from birth to 1 year, and measured sex hormones, gonadotropins, and SHBG. This study was designed to develop methods, assess feasibility, and give information on the time course of the hormones and the correlation structure from the different sample matrices collected in infants.

## Results

The unadjusted geometric mean concentrations of estradiol, estrone, testosterone, LH, FSH and SHBG by matrix and sex are show in Table 1. Generally, statistically significant difference in analytes’ concentrations between feeding regimens was found neither in boys nor in girls, except for lower estradiol level in soy formula-fed boys than in cow formula-fed boys found in both urine and saliva samples (12.60 vs 17.89 pg/mL, p = 0.013 and 13.90 vs 19.66 pg/mL; p = 0.008, respectively), lower LH level in soy formula-fed boys than in cow formula-fed boys found in both urine and saliva samples (0.32 vs 0.55 mIU/mL, p = 0.025 and 0.57 vs 0.85 mIU/mL, p = 0.033, respectively), and higher LH level in soy formula-fed girls than in cow formula-fed girls (0.62 vs 0.36 mIU/mL; p = 0.006) found only in urine samples (Table 1).

**Table 1:** Geometric mean (95% CI) concentrations of urine, saliva and blood analytes by feeding method and sex.

Matrix	Sex	Analyte	Feeding method		
			Breast milk	Cow formula	Soy formula
Urine	Boy	Estradiol (pg/mL)	15.44 (12.82, 18.60), n = 65	17.89 (15.52, 20.63), n = 61	12.60 (10.28, 15.44), n = 59
		Estrone (pg/mL)	87.20 (70.96, 107.14), n = 65	107.80 (89.13, 130.39), n = 64	100.96 (75.10, 135.74), n = 62
		Testosterone (ng/dL)	4.05 (3.43, 4.79), n = 65	3.67 (3.08, 4.38), n = 61	4.32 (3.73, 5.01), n = 59
		LH (mIU/mL)	0.35 (0.26, 0.47), n = 65	0.55 (0.43, 0.70), n = 64	0.32 (0.24, 0.43), n = 62
		FSH (mIU/mL)	1.42 (1.12, 1.78), n = 65	1.11 (0.82, 1.50), n = 64	0.99 (0.76, 1.29), n = 62
		SHBG (nmol/L)	29.57 (24.35, 35.92), n = 65	22.81 (18.95, 27.47), n = 64	25.94 (21.07, 31.96), n = 62
	Girl	Estradiol (pg/mL)	13.77 (11.88, 15.95), n = 61	14.33 (12.41, 16.55), n = 64	17.30 (14.41, 20.77), n = 61
		Estrone (pg/mL)	65.79 (48.48, 89.28), n = 63	94.64 (77.08, 116.20), n = 64	99.77 (75.38, 132.06), n = 63
		Testosterone (ng/dL)	3.43 (2.90, 4.05), n = 61	3.48 (3.02, 3.99), n = 64	2.61 (2.07, 3.30), n = 61
		LH (mIU/mL)	0.47 (0.36, 0.63), n = 63	0.36 (0.28, 0.47) n = 64	0.62 (0.47, 0.82) n = 63
		FSH (mIU/mL)	1.25 (0.95, 1.64), n = 63	1.08 (0.86, 1.36), n = 64	1.50 (1.15, 1.95), n = 63
		SHBG (nmol/L)	26.68 (21.33, 33.38), n = 63	26.76 (21.84, 32.79), n = 64	28.91 (23.39, 35.74), n = 63
Saliva	Boy	Estradiol (pg/mL)	16.66 (13.78, 20.14), n = 61	19.66 (17.31, 22.32), n = 58	13.90 (11.41, 16.94), n = 54
		Estrone (pg/mL)	87.62 (71.92, 106.76), n = 61	114.15 (94.17, 138.37), n = 61	109.79 (81.15, 148.54), n = 57
		Testosterone (ng/dL)	4.99 (4.27, 5.83), n = 61	4.79 (4.06, 5.67), n = 58	5.18 (4.41, 6.09), n = 54
		LH (mIU/mL)	0.77 (0.64, 0.89), n = 61	0.85 (0.72, 1.00), n = 61	0.57 (0.45, 0.71), n = 57
		FSH (mIU/mL)	2.02 (1.71, 2.39), n = 61	1.62 (1.25, 2.09), n = 61	1.51 (1.18, 1.93), n = 57
		SHBG (nmol/L)	33.60 (27.52, 41.01), n = 61	26.92 (22.26, 32.57), n = 61	30.65 (24.87, 37.77), n = 57
	Girl	Estradiol (pg/mL)	15.38 (13.37, 17.70), n = 57	16.52 (14.22, 19.20), n = 59	18.75 (15.85, 22.17)
		Estrone (pg/mL)	63.48 (47.05, 85.64), n = 59	101.77 (84.38, 122.75), n = 59	101.73 (78.84, 132.11), n = 62
		Testosterone (ng/dL)	4.35 (3.72, 5.08), n = 57	4.22 (3.70, 4.80), n = 59	3.20 (2.61, 3.92), n = 60
		LH (mIU/mL)	0.79 (0.64, 0.96), n = 59	0.70 (0.59, 0.83), n = 59	0.95 (0.80, 1.13), n = 62
		FSH (mIU/mL)	1.76 (1.46, 2.14), n = 59	1.52 (1.28, 1.80), n = 59	1.94 (1.57, 2.40), n = 62
		SHBG (nmol/L)	34.14 (27.44, 42.48), n = 59	28.74 (23.12, 35.73), n = 59	34.97 (28.76, 42.51), n = 62
Blood	Boy	Estradiol (pg/mL)	29.32 (22.13, 38.85), n = 8	20.83 (15.90, 27.30), n = 15	22.39 (16.92, 29.65), n = 19
		Estrone (pg/mL)	316.99 (216.49, 464.15), n = 8	206.13 (145.50, 292.01), n = 18	284.11 (187, 12, 431.38), n = 22
		Testosterone (ng/dL)	2.96 (1.72, 5.10), n = 8	4.17 (3.00, 5.80), n = 15	4.47 (3.39, 5.90), n = 19
		LH (mIU/mL)	0.85 (0.50, 1.43), n = 8	1.15 (0.86, 1.52), n = 18	0.94 (0.72, 1.22), n = 22
		FSH (mIU/mL)	2.42 (1.37, 4.27), n = 8	1.99 (1.26, 3.13), n = 18	2.17 (1.61, 2.94), n = 22
		SHBG (nmol/L)	17.60 (12.57, 24.63), n = 8	22.99 (17.80, 29.70), n = 18	25.00 (21.38, 29.22), n = 22
	Girl	Estradiol (pg/mL)	20.13 (16.01, 25.31), n = 13	18.59 (15.19, 22.74), n = 17	24.93 (14.52, 42.79), n = 6
		Estrone (pg/mL)	184.89 (146.92, 232.68), n = 15	171.15 (134.52, 217.75), n = 17	333.20 (186.46, 595.40), n = 8
		Testosterone (ng/dL)	3.90 (2.85, 5.33), n = 13	3.49 (2.74, 4.46), n = 17	3.12 (1.22, 7.95), n = 6
		LH (mIU/mL)	1.13 (0.81, 1.58), n = 15	1.11 (0.92, 1.34), n = 17	1.44 (0.99, 2.08), n = 8
		FSH (mIU/mL)	2.06 (1.23, 3.43), n = 15	1.68 (1.12, 2.53), n = 17	2.22 (1.10, 4.49), n = 8
		SHBG (nmol/L)	25.48 (18.39, 35.29), n = 15	21.92 (16.64, 28.89), n = 17	24.23 (16.36, 35.88), n = 8

CI: confidence interval.

We found strong correlations between measurements from ELISA and from recycling immunoaffinity chromatography (RIC) for all analytes in all matrices. *R* ^2^ values in all 18 correlations (six analytes in three matrices) exceeded 0.85 and 13 of 18 correlations exceeded 0.90. The intra- and inter-assay coefficients of variations (CVs) of all analytes for all matrices are less than 5%. Pair-wise Spearman’s correlation coefficients among urine, saliva and blood samples were generally high (Table 2). All three sex hormones (estradiol, estrone and testosterone) and FSH showed strong correlations among the three matrices (Spearman’s *r* > 0.9). LH had good correlations between urine and saliva samples (Spearman’s r > 0.8) but only moderate correlations between urine or saliva and blood (Spearman’s r between 0.6 and 0.8). Even SHBG, usually measured in serum, showed a strong correlation between urine and saliva samples (Spearman’s r > 0.9) and moderate correlation between urine or saliva and blood samples (Spearman’s r between 0.5 and 0.8). Because strong correlations existed among the matrices and we had urine samples for all visits, the following analyses were based on urine samples.

**Table 2:** Correlations between analyte concentrations in urine, saliva and blood samples.

	Spearman correlation coefficient r (n) between		
	Urine and Saliva	Urine and Blood	Saliva and Blood
Boys			
Estradiol	0.96 (173)	0.98 (42)	0.97 (32)
Estrone	0.96 (179)	0.90 (48)	0.93 (38)
Testosterone	0.93 (173)	0.90 (42)	0.93 (32)
LH	0.82 (179)	0.70 (48)	0.78 (38)
FSH	0.95 (179)	0.84 (48)	0.86 (38)
SHBG	0.93 (179)	0.49 (48)	0.65 (38)
Girls			
Estradiol	0.93 (176)	0.91 (35)	0.91 (26)
Estrone	0.95 (180)	0.89 (39)	0.88 (30)
Testosterone	0.91 (176)	0.92 (35)	0.95 (26)
LH	0.85 (180)	0.67 (39)	0.66 (30)
FSH	0.92 (180)	0.90 (39)	0.94 (30)
SHBG	0.94 (180)	0.67 (39)	0.78 (30)

**Figure 1:**
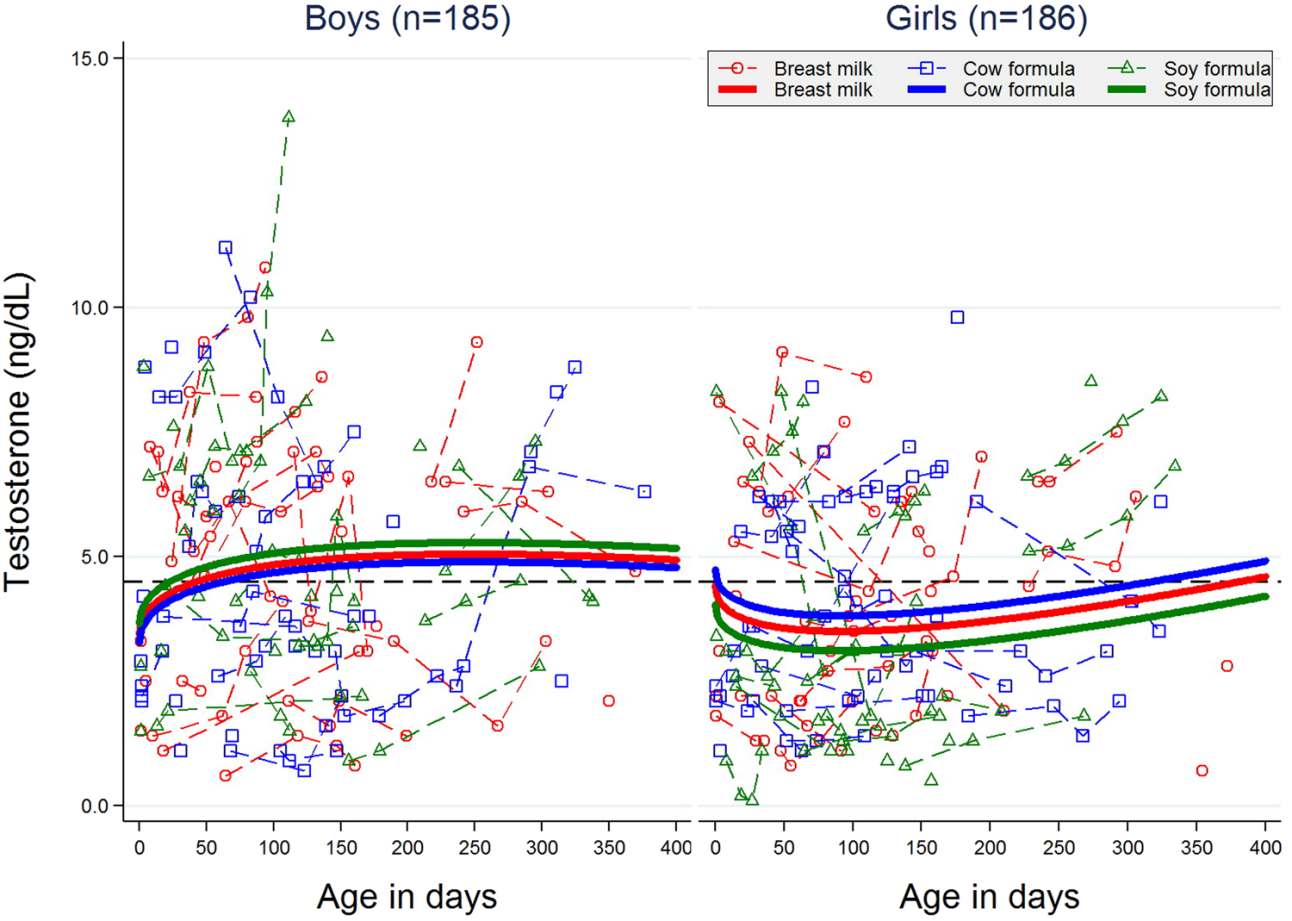
Urinary testosterone concentration (ng/dL) as a function of age (d). Plotted points are individual sample values, and those connected by line segments represent multiple visits by the same infant. Plotted nonlinear trajectories were fitted using generalized mixed model for individual sample values (2 values < 0.5 were excluded).

**Figure 2:**
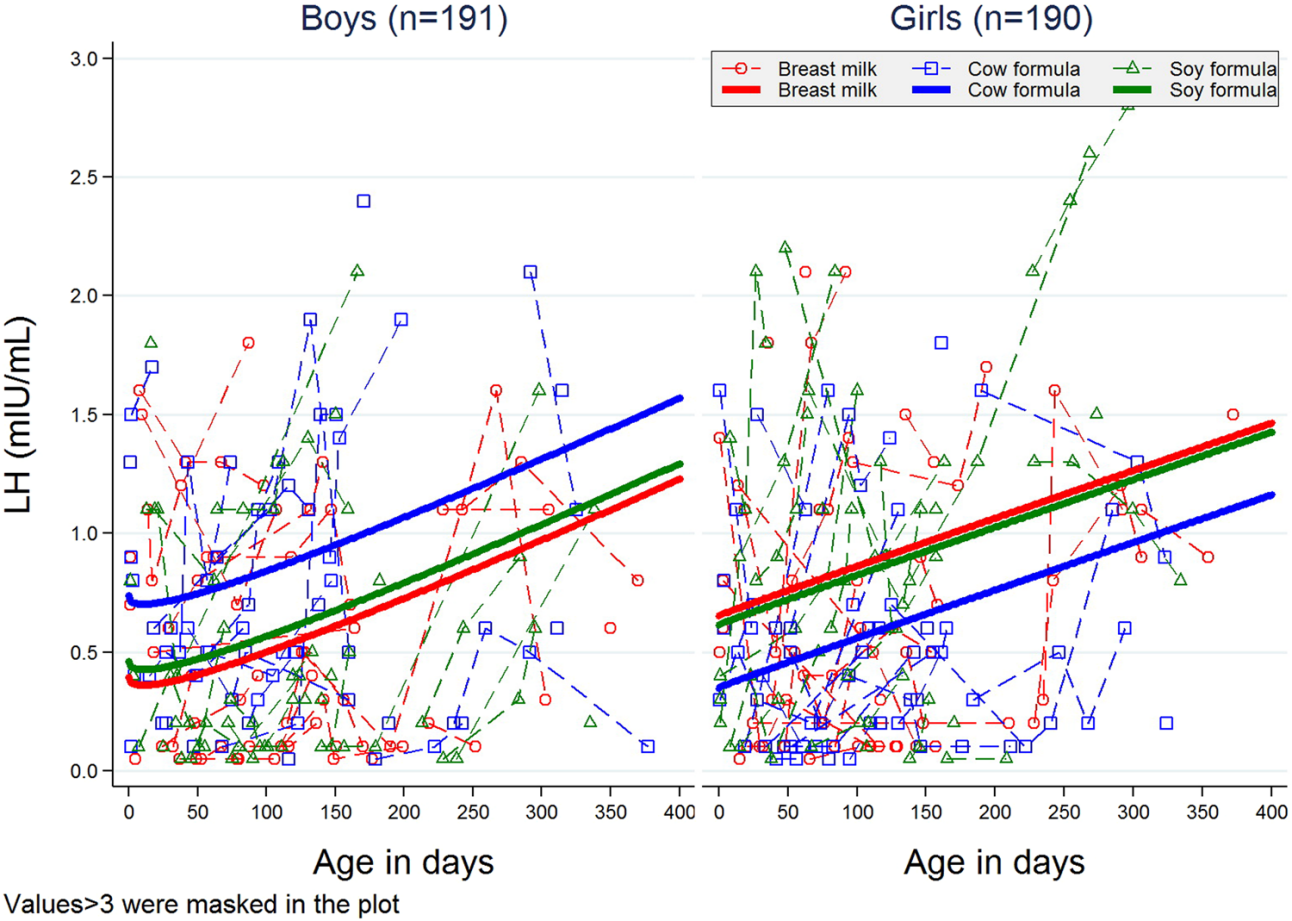
Urinary LH concentration (mIU/mL) as a function of age (d). Plotted points are individual sample values, and those connected by line segments represent multiple visits by the same infant. Plotted nonlinear trajectories were fitted using generalized mixed model for log transformed individual sample values.

In general, variance among subjects was larger than variance among visits by the same subject, though the relative magnitude of these variance components differed among analytes. For example, for testosterone, the within-subject variance was about 25% of the among-subject variance (Fig. 1) whereas for LH, the corresponding proportion was about 66% (Fig. 2).

Although we saw some evidence of heterogeneity in temporal trajectories of analytes concentrations from birth to 1 year across feeding regimens, we detected no statistically significant effects of feeding method on any of the analytes. In 12 sex- and analyte-specific linear mixed models that compared the difference in analyte concentrations between feeding regimens adjusting for race, weight, length, and head circumference, 10 had p-value > 0.30 and remaining 2 had p-value > 0.10.

In particular, we failed (p = 0.37) to confirm a previous finding in marmosets of suppressed testosterone in males on soy formula^20^. We did, however, observe that testosterone trajectories for boys and girls were different both in slopes and intercepts (p = 0.0056 in a 2-degrees-of-freedom test). Although boys had higher testosterone but the slopes appeared flatter with age (Fig. 1). LH appeared lower in breast-milk-fed and soy-formula-fed boys than in cow-formula-fed boys, adjusting for race, weight, length, and head circumference; whereas it appeared higher in breast-milk-fed and soy-fed girls compared to cow-formula-fed girls, however, both the differences were small and not statistically significant (Fig. 2). FSH, Estradiol, estrone and SHBG were not statistically different between either sexes or feeding regimens (data not shown).

Since no statistically significant difference in analyte concentrations was found between feeding regimens, we combined three feeding regimes together. Sex-specific monthly average concentrations of the six analytes tracked the trajectories fitted for the individual measurements well. Most of the analyst increased with age within 1 year except for flat trend was found for SHBG in both sexes and for testosterone in girls (Supplemental Fig. 1). The adjusted monthly average concentrations of the analytes were within typical reference ranges^21, 22^.

## Discussion

It has become a concern that there might be both immediate and delayed consequences when infants are exposed to hormonally active agents in the environment and in their diet. Andres and Gilchrist *et al*. investigated the relationship between breast milk, cow-formula or soy-formula feeding and infants’ development and reproductive organ size in a child cohort from 3 months to 5 years, and no feeding effects were found on reproductive organs volumes and developmental status (mental, motor, and language) in the series of studies^23–25^. However, no hormone levels were reported in these studies and feasible methods to study hormones in infants are awaited.

In this study, we found that the feeding methods had no significant effect on any of the analytes. This result is seemingly different from previous finding in male marmosets of suppressed testosterone on soy formula^26^. Although we did see possible suppression of testosterone in girls fed soy. This result is unexpected and needs further exploration. The reasons may involve but not limited to the following facts. First, marmosets harbor bacteria that convert daidzein to the more potent equol, but human infants do not have those bacteria, and equol was only detected in 35 urine samples. Second, it has been demonstrated that the diets lead to different hormonal responses among different species^27^. Third, among the many homeostatic negative feedback control mechanisms, the central nervous system has the greatest influence to confine gonadotropin-releasing hormone in the so-called “juvenile pause” status in infants. More specifically, the hypothalamic–pituitary–gonadal axis (HPG axis) plays a critical part in the negative feedback control mechanisms by sensitively responding to the sex hormone levels in the peripheral blood of the infants, so as to maintain the sex hormones and gonadotropins at a rather low level in the urine, saliva and blood, despite of any significant effects from the diets^28^. Therefore, feeding methods had limited effects on the hormone levels as shown in the present study. Interestingly, the data showed heterogeneity in temporal trajectories of analytes concentrations. Moreover, the variance among subjects was larger than that among visits by the same subject, and the relative magnitude of these variance components differed among analytes. This may be partly explained by the different effects to the HPG axis by genetic factors, environmental changes, physiological statuses and leptin levels, *etc* ^28^.

Generally, the adjusted monthly average concentrations of the analytes were in accordance with typical reference ranges^21, 22^. However, sex-specific testosterone trajectories were noted in the present study. More specifically, boys and girls were different both in slopes and intercepts, and boys had higher testosterone levels and flatter slopes with age. The reasons may include but not limited to the fact that boys have a testosterone surge during the first few months of life, when testosterone level may be as high as that of an adult male^29^, but the surge disappears later on.

Early life exposure to an exogenous estrogen might have both immediate and delayed consequences. During this period, the infant is thought to be programmed to express male characteristics after puberty, not only in sexual development, but also in setting patterns in the brain characteristic of male behavior^30, 31^. In monkeys, deficiency of male hormones impairs learning and the ability to perform visual discrimination tasks – such as would be required for reading – and retards the development of spatial perception, which is normally more acute in men than in women^32, 33^. In a marmoset model, feeding soy formula to infant male monkeys transiently reduced circulating testosterone by half, and juvenile animals still had evidence of Leydig cell hypertrophy^26^. Girls synthesize estrogen over the first 18 months to two years. An exogenous estrogen might reduce the synthesis of endogenous hormone in either sex, interfering with long term programming^34, 35^.

For this study, we wanted to characterize a broad array of hormones. To do so, we used a micro-scale system which employed an array of capillary immunoaffinity columns as the isolation step coupled with laser-induced fluorescence detection of the isolated analytes. It is capable of measuring up to 30 different analytes in a 10-μl sample simultaneously. Comparisons in values obtained by this method and conventional high-sensitivity ELISA assays were very similar (*R* ^2^ values are in the range of 0.92–0.99)^36^. We found most concentrations are in the conventional normal range. We found that estradiol range was high, but there are few reference values in the literature for infants less than one year of age, and the identity was confirmed with mass spectrometry. The high precision and low variation of the assays in the study suggest that this new technique for analyzing multiple analytes in a single, small-volume sample may be the only way to perform this kind of study.

Collecting specimens from relatively large numbers of small children requires compromise. Many analytes are secreted intermittently or with a diurnal peak. Twenty-four hour urine collection, indwelling blood sampling devices, and a bed in a metabolic ward would be optimal, but are not practical in the real world. The samples in the study were collected at approximately the same time of day, in the same order, and at least one hour after feeding. We thus could see general trends in concentrations of hormones but may miss any but gross effects on patterns or peaks of synthesis or excretion. We saw strong correlations among the sample matrices, leading us to believe that, while the kinetics may differ among the matrices, most of the information is obtained by analysis of one or two.

Our methods of sample collection, processing, and analysis appeared suitable for use in longitudinal research. Our prior belief was that saliva collection would always be easier than blood collection. We found, however, that in very young children, especially those breastfed, 2 ml of saliva can be difficult to collect without distressing the child, and the effects of oral hygiene might affect saliva hormonal levels in the infants, we therefore think urine samples are the most easily harvested, so they are predominantly used to present full time-course data for testosterone levels in this study. On another hand, modern ultra-sharp lancets and sweet suckers made collection of the small amounts of blood needed by the micro methods quite tolerable. It would seem reasonable to substitute blood collection for saliva collection in young infants if two matrices are needed.

This study was a pilot. Although we analyzed about 800 samples, the effective sample size for inference about feeding method is small. Given that, we did not see a strong uniform depression of endogenous sex hormones and gonadotropins in human infants fed a soy-formula diet. Our data cannot rule out more complex dietary differences that vary with age. Because oral hygiene habits determine the amount and types of bacteria harbored in the oral cavity which may have potential effects on hormone levels in adults^37^, the effects of oral hygiene on salivary hormonal levels in the infants may need further evaluation in future studies. In addition, socioeconomic status (SES) and maternal demographics might have impacts on infant hormone concentrations given that we know that SES and genetic factors often are related to all health outcomes. They might be major predictors of child hormone concentrations. However, no SES and maternal demographic data were available in our study and we hope the studies in the future may fill the information gap. A potential limitation in terms of studied outcomes is that we did not examine estrogen stimulation of reproductive tissues in girls. This is because Andres and Gilchrist *et al*. have already provided convincing evidence showing no feeding effect in reproductive organ volumes and developmental status in the serial studies^23–25^, so we didn’t study inappropriate estrogen stimulation of reproductive tissues in girls in current study. However, further research is needed on potential effects for the reproductive tissues that might be related to sex hormones in early life. Moreover, the urinary testosterone data fails to show any evidence for rise in testosterone levels in boys during the neonatal period, with a decline beyond 3–4 months, as well as a male-female difference in testosterone levels during this period. Therefore, urinary testosterone levels may not accurately reflect blood levels during mini-puberty, and further studies are needed to clarify to what extent the urinary testosterone levels can accurately reflect blood levels during this age-specific window.

The possibility that exogenous substances, such as trace amounts of environmental pollutants or dietary components such as isoflavones, could have hormonal effects in humans is known as the “endocrine disrupter” hypothesis. In 1996, the U.S. Congress enacted two pieces of legislation requiring the US Environmental Protection Agency to screen and test chemicals in food (Food Quality Protection Act of 1996) and water (Safe Water Drinking Act Amendments of 1996) for estrogenic and possibly other hormonal activity^38^ in the hopes of preventing such exposures. In a 1999 consideration of this topic, the National Research Council ranked isoflavone exposure as the highest (in the general population) of all putative endocrine disrupting compounds^39^. Thus, infants whose diets consist of 100% soy formula are a model group, and any method that fails to find effects in them would be unlikely to detect effects from other agents. We examined the nonlinear time trend of hormone levels by feeding methods (see Figs 1 and 2) and wanted to know if there is a specific threshold/time point where the hormone levels had a significant change. However, no statistically significant threshold was found. The reason might be the relatively small sample size and sparse number of visits (up to four visits for each infant) during the study.

In conclusion, although our study shows no difference in urinary testosterone levels between soy-fed boys and other boys and we cannot resolve whether or not soy feeding might interfere with testosterone levels, possibly due the methodological limitations. We demonstrated that blood, urine, and saliva samples are readily collectible and suitable for some multi-hormone analyses from infants, and the methods may allow direct examination of hypotheses concerning endocrine effects of environmental or dietary compounds in infants. We believe the presented method could allow facilitate powerful and specific investigations of the endocrine disrupters than had been possible before, and could be useful to investigate hormonally related phenomena in small children.

## Methods

### Study Design

We used the data from the Study of Estrogen Activity and Development (SEAD). The detailed description of the SEAD was published elsewhere^19^. Briefly, SEAD was conducted between 2006 and 2009 at the Children’s Hospital of Philadelphia (CHOP), the Hospital of the University of Pennsylvania (HUP), and affiliated clinics, with the laboratory assays done at Division of Laboratory Sciences of the U.S. Centers for Disease Control of Prevention (CDC) and the Ultramicro Analytical Immunochemistry Resource of the U.S. National Institutes of Health (NIH). The Institutional Review Boards at CHOP, HUP, and the U.S. National Institute of Environmental Health Sciences (NIEHS) approved the study. All methods in the study were performed in accordance with the relevant guidelines and regulations of the aforementioned institutions.

The study recruited children from the nursery at HUP, the clinics at CHOP, and several CHOP satellite clinics. The researchers used flyers, information sessions targeting the clinic staff, and a computer-generated reminder to physicians when they accessed a potentially eligible patient’s record. Children were eligible if they had been born at term (37–41 weeks), with birth weight 2500–4500 g, met one of the feeding regimens and ages (Table 3), and had no major illness or birth defect. Exclusion criteria included chromosomal anomaly, major malformation, or any endocrinopathy (ambiguous genitalia, congenital hypothyroidism, *etc*.). Families were compensated for meal and travel expenses and given coupons for local food stores.

**Table 3:** Feeding regimen specifications.

Ages at examination^a^	Feeding Regimen		
	Breast milk^b^	Cow-milk formula^c,d^	Soy formula^e^
1–15	Breast milk exclusively	Cow-milk formula exclusively	Soy formula exclusively
16–31	Breast milk exclusively or Breast milk and Cow milk formula	Cow-milk formula exclusively	2/3 of lifetime on soy formula exclusively and continuously, including the two weeks prior to the exam

^a^Ages 1–15 are newborn up to 48 hours, 1, 2, 3, 4, 5, 6, 7, 8, 9, 10, 11, 12, 13 and 14 weeks respectively. Ages 16–31 are 15, 16, 17, 18, 19, 20, 21, 22, 23 weeks and 6, 7, 8, 9, 10, 11 and 12 months respectively.

^b^Breast-milk Restriction: A baby in the breast-milk category could not have had any soy foods in his/her lifetime.

^c^Cow-milk formula Restriction: A baby in the cow-milk formula category could not have had any soy foods in his/her lifetime.

^d^Cow-milk formula Exception: If a baby was breast-fed in the nursery, the baby must have gone home on cow-milk formula and have been on cow-milk formula exclusively ever since. Such a child could not participate until she/he had been fed exclusively cow-milk formula for at least 2 weeks.

^e^Soy-formula Exception: If a baby was fed something other than soy formula in the nursery, the baby must have gone home on soy and been on soy exclusively ever since. Such a child could not participate until s/he had been fed exclusively soy formula for at least 2 weeks.

The signed consent forms were obtained from all the participants for the study participation, use of samples and publication. All the data used in the analysis and publication were de-identified and no personal information was disclosed. No information or images that could lead to identification of a study participant were contained in the manuscript.

For feasibility reasons, we did this study mostly cross-sectionally. Although we did not expect to be able to test hypotheses about differences by feeding method, we wanted to include breast-fed children and children fed both soy and cow milk based formulas in order to inform the planning of a longitudinal study. Since the way infants were fed changed over the course of their first year of life, we set feeding regimens that would provide substantial contrast in the feeding histories of the participants without making recruitment too difficult. The definitions of the feeding regimens were described in detail elsewhere^19^. In brief, infants in the breast milk, cow formula and soy formula groups were exclusively fed by breast milk, cow formula or soy formula within three months of age. Breast-milk-fed and cow-formula-fed infants could have cow formula or breast milk exclusively or together after three months but were not allowed to have had any foods containing soy in their lifetime. For infants in cow formula or soy formula groups, if a baby was breastfed or cow-formula-fed in the nursery, the baby must have gone home on cow formula or soy formula and have been on cow formula or soy formula exclusively ever since. Such a child could not participate until he/she had been fed exclusively cow formula or soy formula for at least 2 weeks. The feeding methods were recorded at the beginning and throughout the study. A given child was allowed to be in the study for up to 4 visits, so long as they met age and feeding requirements. The requirements were described in detail elsewhere^19^. All decisions regarding infant feeding were made by families in consultation with their own physicians. The study called for 372 total visits: 2 boys and 2 girls in each of 31 ages (<48 hours of age, at weekly intervals from 1 week to 23 weeks of age, then at monthly intervals from 6 months to 12 months) and three feeding regimens.

### Sample Collection and Laboratory Methods

The researchers mailed the parents a special gel-free cotton blend diaper which they were to put on the infant. After the overnight, diaper was removed in the morning of the clinic visit. The diaper was checked in the clinic, and if it was wet, it was removed and placed in a 50 cc syringe and compressed. If the diaper was badly soiled or 5 cc of urine could not be collected, then girls were re-diapered and boys were bagged. A saliva sample was collected at least 60 minutes after a feeding. If residual formula/breast milk was present, the child’s mouth was swabbed with a sterile 2 × 2 gauze pad. The saliva collection device was made at the NIH clinical center. It was a vacuum device with a soft tube that was placed on the side of the infant’s mouth or under the tongue^40^. The researchers collected 2 mL of saliva per child. Because of the difficulty of collecting blood from small children, the study planned to focus on urine and saliva as the primary sample matrices, and attempted to collect both from all children at each visit. For validation purposes, blood samples were from one boy and one girl in each age interval. Capillary blood was collected between 30 and 120 minutes after a morning feeding by a heel stick, and was filled four circles on two Guthrie cards.

In total, urine samples were collected in 381 visits (9 more than planned, because of some inadvertent extra scheduling) from 84 boys and 82 girls aged from birth to 12 months (Supplemental Figure 2, panel A), saliva samples in 359 visits (missing mostly newborns, Supplemental Figure 2, panel B), and blood samples in 88 visits (Supplemental Figure 2, panel C).

All the samples were transported to the CHOP’s General Clinic Research Center (GCRC) and frozen and stored in sterile cryotubes at −70 °C in freezer. For analyzing, the samples were thawed, divided into aliquots, and shipped to the aforementioned laboratories.

Urine samples were analyzed at the Division of Laboratory Sciences of CDC. The automated online solid-phase extraction (SPE) coupled to isotope dilution high-performance liquid chromatography-tandem mass spectrometry (HPLC-MS/MS) was used for measuring estradiol, estrone, testosterone, luteinizing hormone (LH), follicle-stimulating hormone (FSH) and SHBG. Briefly, the analytes of interested were enzymatically hydrolyzed using *β*-glucuronidase/sulfatase (Helix pomatia, H1). After hydrolysis, the analytes were preconcentrated by online SPE, separated by reversed-phase HPLC, and detected by isotope dilution atmospheric pressure chemical ionization-MS/MS. The SPE recoveries were 83–94%, and the coefficients of variation were 4–12%. Details of the method and its validation were reported elsewhere^41^.

Blood and saliva samples were analyzed at the lab of NIH by recycling immunoaffinity chromatography (RIC) using an array of capillary immunoaffinity columns packed with antibody-coated glass beads. Each column contained a single, specific antibody and isolated its specific analyte, allowing the sample to pass to the next column. In this way, all analytes could be isolated from the same sample during the same run. The specificity of each antibody was immunochemically checked by 2-dimensional Western blotting, against all of the analytes of interest to ensure no cross-reactivity, prior to use. Bound analytes were labeled with laser dye and detected by laser-induced fluorescence using a scanning detector and a fiber-optic spectrometer. The concentrations of each analyte were calculated by comparison with standard curves constructed by running known amounts of each analyte through the array under the same conditions. Additionally, the analytes from each column were collected and subjected to characterization by mass spectrometry to ensure specificity.

To validate the RIC assays against ELISA, the NIH lab made triplicate runs of spiked blood spots, urine samples, and saliva samples for all analytes and calculated R^2^ values from linear regression analyses using GraphPad 4 software^42^. To assess reproducibility of the RIC assays, we calculated intra- and inter-assay coefficients of variations (CVs) from data obtained by running the same sample 5 times within the same day and on 5 consecutive days. The method was described in detail elsewhere^36^.

All samples had all analytes detectable, except for 27 urine LH determinations, 5 urine and 5 saliva FSH determinations.

### Data analysis

This was a primarily descriptive study of children at ages when hormone levels, measured in multiple matrices, were changing. Thus the primary analytical approaches involved the correlation structure of the analyte concentrations in different matrices, the graphical display of the analyte trajectories through time, and fitting of appropriate regression models. Hormone concentrations are all continuous variables. We transformed them using natural logarithmic transformation when necessary to achieve symmetric approximately normal distributions of regression residuals, which in turn increased the validity of estimated confidence intervals. Residuals for all hormones were more symmetric after transformation, and we presented the analyses based on the transformed values. Since few samples reported as below the limit of detection (LOD), we used listwise deletion method for missing data^43^.

In adults, where the concentration of a urine specimen can vary greatly and creatinine production does not vary strongly with age, creatinine correction removes variability due to differences in urine concentration. Children, especially infants, have relatively less ability to concentrate urine. In addition, creatinine production increases with lean body mass whereas urine production increases less steeply and there is thus a non-linear increase in creatinine concentration with age^44^. Using the standard method for creatinine correction would then produce a negative slope in age for an analyte that was present at a constant concentration over the first year. We have thus chosen to present results from analysis of urine without creatinine correction.

Unadjusted average concentrations of analytes between feeding regimens by sexes were compared using Kruskal-Wallis H test and Bonferroni-adjusted p-values were used for post-hoc multiple comparison^45^. We further used locally weighted linear regression method to explore the temporal trajectories of the various hormones by sex and feeding method^46^. For inference, we used linear mixed models to account for possible correlations among hormone levels from multiple visits of the same infant. We fitted liner mixed regression models that included feeding regimen as fixed effect with separate intercepts and a common slope with respect to age, adjusting for race, weight, length, and head circumference^47^. The models accounted for inter-infant differences in hormone levels via random infant-specific intercepts. Because hormone levels are sex-specific and potential estrogenic effects may be different in boys and girls, data were modeled separately by sex. Monthly average concentrations of the analytes were estimated from the linear mixed models controlled for the race, weight, length, and head circumference of the infants.

The statistical analyses were performed using Stata version 12 and SAS version 9.13^48, 49^. All tests were two-tailed and a p-value less than 0.05 was considered statistically significant.

## Electronic supplementary material

Supplemental Figures
