# Supplementary material for: Sex Hormones, Gonadotropins, and Sex Hormone-binding Globulin in Infants Fed Breast Milk, Cow Milk Formula, or Soy Formula
Source: Sci Rep. 2017 Jun 28;7:4332. doi: 10.1038/s41598-017-04610-y (PMC5489524; doi:10.1038/s41598-017-04610-y)
Supplement: Supplementary file 1 — Supplemental Figures [file 41598_2017_4610_MOESM1_ESM.pdf]

Supplementary information:

Sex Hormones, Gonadotropins, and Sex Hormone-binding Globulin in Infants Fed Breast Milk, Cow Milk Formula, or Soy Formula

Xin Fang [1], Lei Wang [2], Chunhua Wu [3,4], Huijing Shi [3,4], Zhijun Zhou [3,4], Scott Montgomery [5,6,7], Yang Cao [1,5]

[1]Unit of Biostatistics, Institute of Environmental Medicine, Karolinska Institutet, Stockholm 17177, Sweden. [2]Department of Oral & Maxillofacial-Head & Neck Oncology, the Ninth People's Hospital, Shanghai Jiao Tong University School of Medicine, Shanghai Key Laboratory of Stomatology, Shanghai 200011, China. [3]School of Public Health/Key Laboratory of Public Health Safety of Ministry of Education, Fudan University, Shanghai, 200032, China. [4]Collaborative Innovation Center of Social Risks Governance in Health, Fudan University, Shanghai, 200032, China. [5]Clinical Epidemiology and Biostatistics, School of Medical Sciences, Örebro University, Örebro 70182, Sweden. [6]Clinical Epidemiology Unit, Karolinska University Hospital, Karolinska Institutet, 17177 Stockholm, Sweden. [7]Department of Epidemiology and Public Health, University College London, WC1E 6BT, UK

Correspondence and requests for materials should be addressed to L.W. (wanglei@sh9hospital.org)

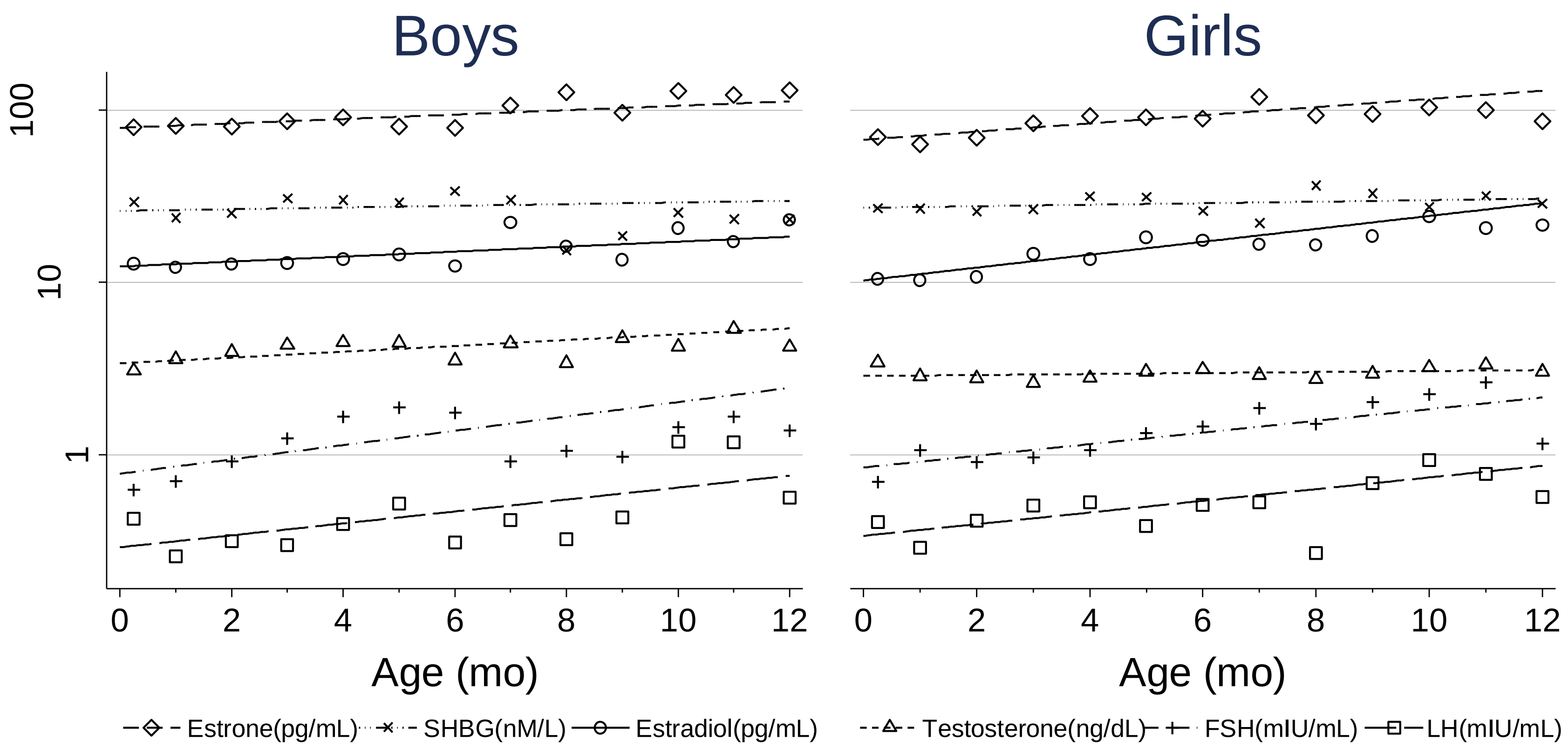

Supplemental Figure 1. Average urinary concentration of sex hormones, gonadotropins and SHBG in boys and girls from birth to 1 year of age

Linear trajectories are those fitted by mixed-model regressions to individual sample values. Plotted points are average values estimated from mixed-model analysis of variance that adjusts for each infant not being seen in every age.

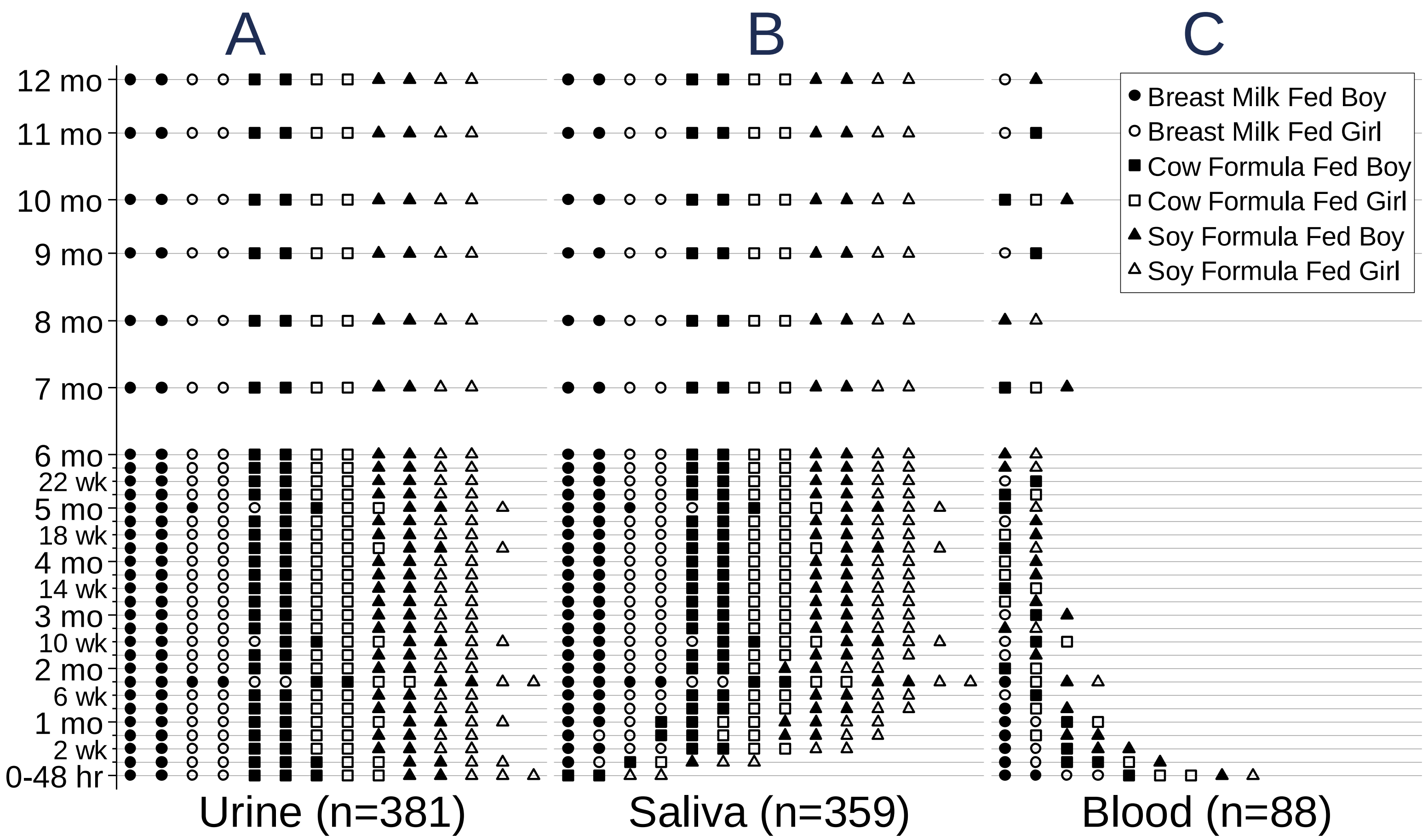

Supplemental Figure 2. Sample allocation to age interval, feeding method, and sex for each sample matrix
